# Comparison of Nerve Growth Factor with Fetal Bovine Serum for Promoting Closure of Defects in Corneal Epithelial Cell Layers

**DOI:** 10.3390/biomedicines14071619

**Published:** 2026-07-18

**Authors:** Michael R. Kozlowski

**Affiliations:** Arizona College of Optometry (AZCOPT), Midwestern University, 19555 North 59th Avenue, Glendale, AZ 85308, USA; mkozlo@midwestern.edu; Fax: +1-623-572-3911

**Keywords:** nerve growth factor (NGF), fetal bovine serum (FBS), neurotrophic keratitis (NK), corneal epithelial (CE)

## Abstract

**Background**: Disruption of the corneal epithelial (CE) cell layer is a pathological feature of several ocular disorders including the potentially severe condition, neurotrophic keratitis (NK). The drug, Oxervate™, whose active principle is recombinant human nerve growth factor (NGF), has been shown to promote healing of the corneal epithelial layer in NK. This effect could result from NGF stimulating the repair and regeneration of corneal nerves so that they provide better trophic support for CE healing and general health. Another mechanism of NGF in promoting CE layer healing may be through direct stimulation of the division and migration of CE cells. Fetal bovine serum (FBS) has also been found to promote CE layer healing in experimental studies. Like Oxervate™, FBS contains NGF, but it also contains several other bioactive components including other growth factors. The present study compares the effects of FBS to those of NGF to examine whether the combination of bioactive components in FBS might be more effective than NGF alone in producing corneal wound healing. **Methods**: CE cells of the HCE-S line were grown in 96-well plates fitted with a silicon plug that occluded a 1 mm circular area in the center of each well. When the cells reached confluence, the plugs were removed, resulting in the cell layers each containing a similar central defect. Different amounts of NGF, FBS, and a combination of the two were then added to each well. The effect of these treatments on the amount of closure of the defect after 24 h was measured and compared. **Results**: NGF increased the amount of closure of the defect after 24 h. At a concentration of 250 nM, NGF produced a significant, 25 ± 9% increase in closure. No further increase in effect was seen when the NGF concentration was increased to 500 nM. FBS also produced an increase in the closure. At an FBS concentration of 10%, this increase was 118 ± 27%, which was significantly greater than the percentage increase produced by NGF. The addition of both 250 nM NGF and 10% FBS together produced no greater amount of closure than that produced by FBS alone. **Conclusions**: These findings are consistent with earlier data suggesting that NGF can promote corneal healing through a direct effect on CE cells. They also show that FBS is more effective than NGF alone in producing this effect. Since the therapeutic activity of NGF in NK may be partly mediated through a direct action on corneal epithelial cells, identifying the factors in FBS that promote corneal healing might lead to more effective treatments for NK.

## 1. Introduction

Defects in the epithelial cell layer of the cornea are a part of the pathology of several ocular disorders including the sight-threatening condition, neurotrophic keratitis (NK) [1,2,3]. In cases of NK, disruption of the corneal epithelial (CE) cell layer can progress from punctate epitheliopathy to persistent large defects [1,2,3]. In more severe presentations, these defects can lead to corneal ulceration and even perforation [1,2,3]. These pathological changes are thought to be triggered by the loss or impaired functioning of corneal nerves, resulting in a decrease in their release of trophic factors within the CE cell layer [1,2,3,4]. Common causes of NK, such as herpes simplex or zoster virus infections, ocular surface disease, and corneal surgeries such as keratoplasty are, indeed, associated with corneal nerve damage [1,2,3].

The drug, Oxervate™, whose active principle is recombinant human nerve growth factor (NGF), is effective in the treatment of NK [5,6]. There is evidence that this therapeutic action is mediated, at least in part, by the restorative action of NGF on damaged corneal nerves [2]. NGF has been found to increase both the number and density of nerve fibers in the corneal epithelium in patients with NK [7]. Furthermore, following damage to corneal nerves produced by keratoplasty, the concentration of NGF in the tear film is correlated with the number of regenerating nerve bulbs observed [8,9]. NGF is also one of the trophic factors released by corneal nerves and there is evidence that NGF can directly increase their rate of division and migration of CE cells [2,10,11]. Experimental studies have shown that NGF agonists are also effective in treating ocular surface disorders that have no clear relationship to a loss of corneal innervation such as allergic conjunctivitis and dry eye disease [2]. In addition, NGF has been shown to increase the clonicity, replication and migration of CE cells, as well as to promote the closure of scratch injuries in CE cell layers [2,10,11]. This suggests that NGF may also act directly on CE cells in treating NK. Given the complexity of the interactions between the corneal nerve cells and the CE, both of these mechanisms could also be involved [2,10,11].

Other agents such as amniotic membranes, autologous serum, human umbilical cord serum, platelet-rich plasma, and fetal bovine serum (FBS) have also been found to promote CE healing either in vitro or clinically, including in NK patients [3,8,12,13,14,15]. While each of these substances contains NGF, they also contain many other components, including growth factors other than NGF [3,8,12,13,14]. The present study compares the effects of one of these substances, FBS, to those of NGF in an in vitro model of corneal wound healing to determine whether the combination of bioactive components present in FBS might be more effective than NGF alone.

## 2. Methods

HCE-S cells were obtained from Applied Biological Materials (ABM; Richmond, BC, Canada; Cat. No.T0737) and grown according to the supplier’s instructions. HCE-S is a naturally immortalized human corneal epithelium cell line [16]. Briefly, the cells were cultured in 25 cm^2^ tissue-culture-treated flasks in a combination of PriGrow III medium (ABN, Richmond, BC, Canada) plus 100 IU of penicillin and 100 μg/mL of streptomycin (Fisher Scientific, Waltham, MA, USA), which will be referred to as “basal medium”. For cell expansion and after replating, the basal medium was supplemented with 10% fetal bovine serum (FBS, Fisher Scientific, Waltham, MA, USA), and this medium was replaced twice a week.

After two weeks of growth, the cells were detached from the flasks using trypsin, triturated, and replated in basal medium supplemented with 10% FBS in either new flasks or 96-well plates. The plates used were Oris™ Cell Migration Assay plates (Platypus Technologies LLC, Madison, WI, USA). The cells were seeded in the wells at a density of 6 × 10^5^ cells per well. This overseeding of the wells decreased the time necessary for the cells to reach confluence. Each well was fitted with a silicon plug that occluded a 1 mm diameter circular area in the center of the well. The cells were cultured for 2 days in the plates with the basal medium plus 10% FBS being replaced after 24 h. The plug was maintained in a uniform, circular cell-free zone (defect) in the center of each CE layer as the cells reached confluence.

The plugs were then gently removed from the wells in order not to disturb the cell layers. The medium was replaced with either basal medium, basal medium plus FBS (1% or 10%), basal medium plus NGF (recombinant human NGF, Fisher Scientific, Waltham, MA), basal medium plus the vehicle for NGF (phosphate-buffered saline with 1% bovine serum albumin; PBS-BSA; Sigma-Aldrich, St. Louis, MO, USA), or basal medium plus a combination of both FBS and NGF. The cell layers were cultured for an additional 24 h, fixed with cold 100% ethanol, and stained with crystal violet (Fisher Scientific, Waltham, MA, USA).

**Figure 1:**
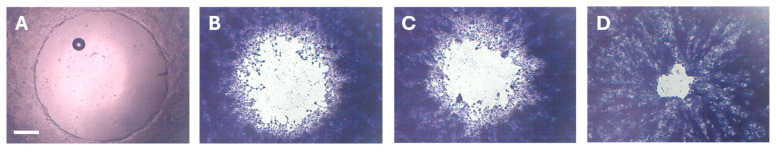
Representative images of defects in CE cell layers. (**A**) Cell layer immediately after removal of the silicon plug. (**B**–**D**) Cell layers 24 h after removal of the plug that had been cultured either without NGF or FBS (**B**), with 250 nM NGF alone (**C**), or with 10% FBS alone (**D**). The cells in panel A are unstained (pink hue is due to indicator in medium) and those in panels (**B**–**D**) are fixed and stained with crystal violet. The bar in panel (**A**) represents 200 μm and all panels are at the same magnification. See Section 2 for more details.

The layers were photographed immediately after removal of the plugs (unstained) and again 24 h later (stained) using a Moticam-5 digital camera attached to a Motic 160 M inverted trinocular microscope at a magnification of 25× (VWR, Radnor, PA, USA) (Figure 1). Defect sizes were then measured in both the pre- and post-treatment photographs using Image-J software 1.54g (https://imagej.net/ij/, accessed on 18 March 2025). The amount of defect closure was measured as the cell-free area after treatment subtracted from the cell-free area prior to treatment. Percent defect closure was calculated as the amount of closure divided by the original defect area expressed as a percentage.

Individual means were compared using *t*-tests (with Dunnett’s test applied to multiple comparisons) and comparisons of the means of three or more groups were made using a one-way analysis of variance (IBM SPSS Statistics 31, IBM; https://www.ibm.com/products/spss-statistics, accessed on 10 January 2026).

## 3. Results

**Figure 2:**
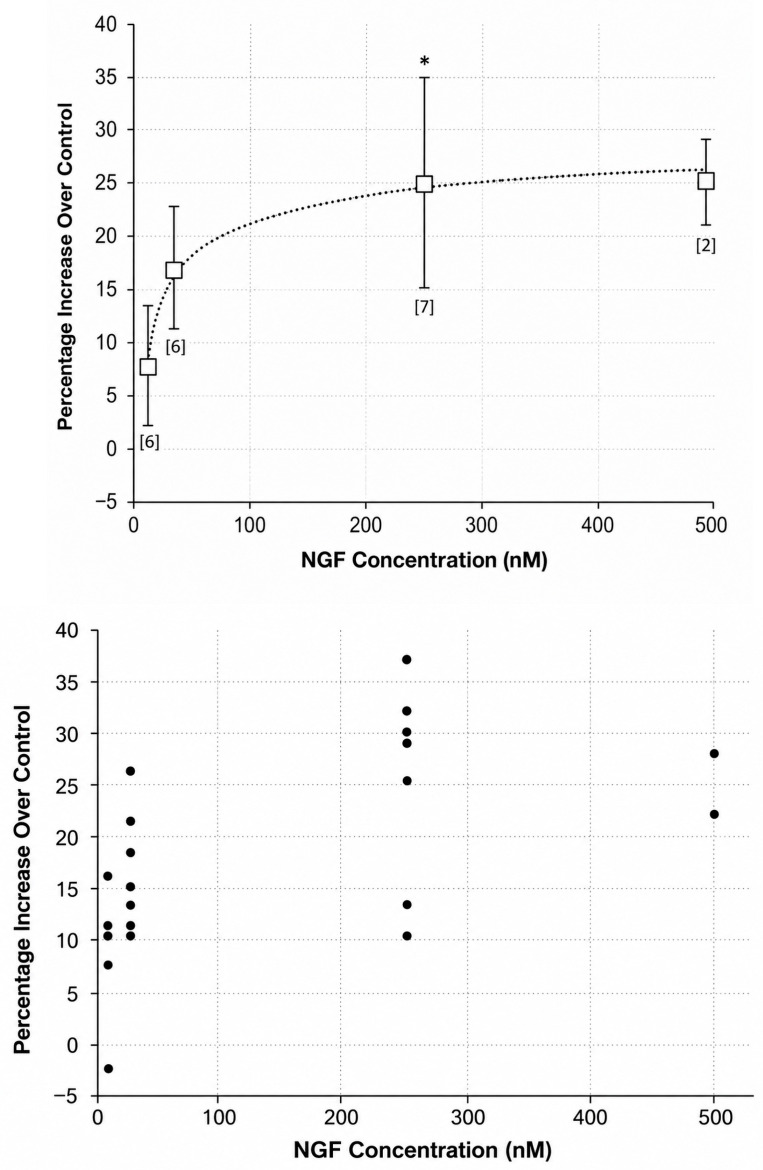
The effect of NGF (2.5 nM, 25 nM, 250 nM and 500 nM) on the amount of closure of defects in CE cell layers grown in basal medium after 24 h of exposure. Values represent percentage change in amount of closure in layers treated with NGF compared to those treated with the vehicle for NGF (control). Data are combined from 7 experiments in which each value was determined in quadruplicate. The top graph shows the mean value and the bottom graph shows individual values. In the top graph, the numbers of replicate experiments used to determine the effect at each concentration are shown in brackets (these numbers may differ from the number of points in the lower graph since some points overlapped). Error bars represent standard deviations. NGF significantly increased the amount of closure in a dose-dependent manner (1-way ANOVA, *p* < 0.01). The absolute amount of closure produced by 250 nM NGF was 51 ± 11%, while that which occurred in vehicle-treated wells was 42 ± 9%. * Significantly greater than 0% NFG (*t*-test, *p* < 0.001).

NGF treatment produced a greater amount of closure of the defects in the CE layers cultured in basal medium than did vehicle treatment after 24 h of exposure (Figure 1C and Figure 2). This increase, which reached 25 ± 9% at the highest NGF concentrations, was statistically significant. In terms of total closure of the defect, the amount of closure with NGF treatment was 51 ± 11%, while that after vehicle treatment was 42 ± 9%. The percentage increase in closure with NGF was dose-dependent and was equal at the highest concentrations used (250 nM and 500 nM), suggesting that it had reached its maximum level.

**Figure 3:**
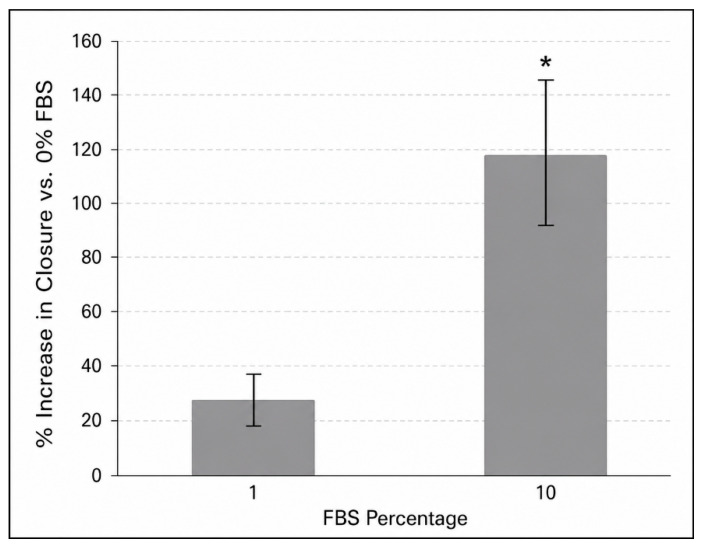
The effect of FBS on the amount of defect closure at 24 h. Cells were grown in basal medium either without FBS (control) or with FBS at the percentages indicated during the treatment period. Values represent the amount of closure with each amount of NGF as a percentage of that after control treatment (no NGF). Addition of FBS significantly increased the amount of closure in a dose-dependent manner (1-way ANOVA, *p* < 0.001). The absolute amount of closure in the absence of FBS (control treatment) was 34 ± 4% and that in the presence of 10% FBS was 75 ± 9%. * Significantly greater than 0% and 1% FBS (Dunnett’s test, *p* < 0.001).

Addition of FBS to the basal medium after removal of the plug also increased the amount of closure in CE layers (Figure 1D and Figure 3). The amount of closure at the highest FBS percentage used (10%) was 118 ± 27% greater than the amount of closure in basal medium alone. This resulted in an absolute amount of closure of 75 ± 9% in the presence of 10% FBS compared to 34 ± 5% in its absence (control treatment).

The effect of FBS was dose-dependent over the range used but was still increasing at the maximum percentage tested. Higher percentages of FBS did not further increase the amount of closure, possibly because they excessively altered the medium and, therefore, affected cell growth. The increase in the actual amount of closure in layers exposed to the highest FBS percentage used was 147% greater than that of layers exposed to a 250 nM concentration of NGF (75 ± 9% versus 51 ± 11%, respectively), which was a statistically significant difference (*t*-test, *p* < 0.001).

**Figure 4:**
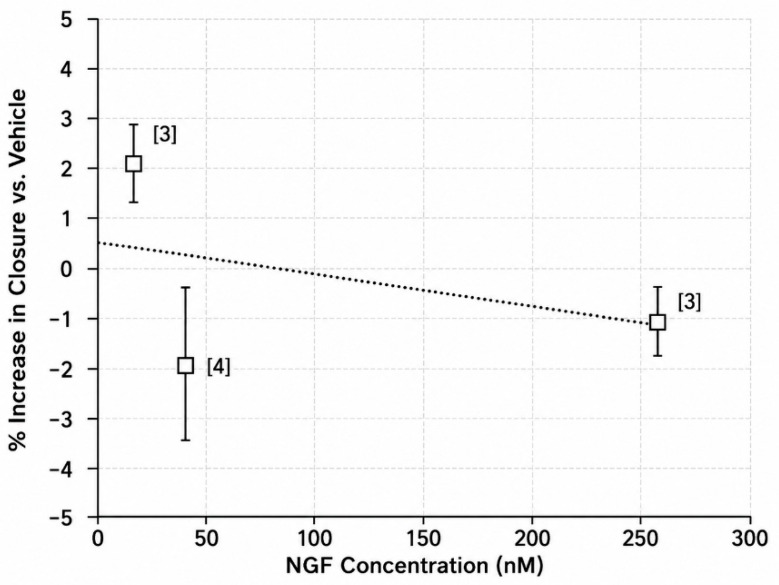
The effect of NGF (2.5 nM, 25 nM, and 250 nM) on the amount of closure of defects in CE cell layers after 24 h of treatment in the presence of 10% FBS. Values represent percentage change in closure in layers treated with 10% FBS plus NGF compared to those treated with 10 FBS plus the vehicle for NGF (control). Data are combined from 5 experiments in which each value was determined in quadruplicate. The numbers of replicate experiments used to determine the effect at each concentration are shown in brackets. Error bars represent standard deviations. Total absolute amount of closure with 10% FBS alone was 86 ± 6%, and that with 10% FBS plus 250 nM NGF was 85 ± 6%. The absolute amount of closure in the control layers was 42 ± 9%.

Adding both FBS (10%) and NGF to the basal medium at the same time produced essentially no greater increase in closure than FBS alone (Figure 4). This suggests that the effect of NGF alone and that of FBS (which also contains NGF) are not additive.

## 4. Conclusions

The present study employed an in vitro system to model the healing of defects in corneal epithelial layers. This system used a human CE cell line (HCE-S) which had naturally immortalized and which shares many of the properties of native CE cells [16]. Defects were produced in the layers that were circular and 1 mm in diameter, rather than the thin lines seen in the “scratch” assays used in other studies [8,17]. This larger, circular shape more closely mimics the geometry of the CE lesions seen in NK and, because of its larger cross-section, also takes more time to heal [18]. The more protracted healing time may permit two of the important processes involved in healing, cell migration and cell proliferation, to both occur, whereas only the more rapid cell migration process is likely to occur in the healing of a scratch assay [18].

The results of this study support the idea that NGF can increase the closure of defects in CE layers through a direct effect on CE cells [2,8,12,13,14]. Thus, it is possible that the ability of Oxervate™ to heal corneal defects in vivo may also be due, in part, to this effect [8,12,13,14]. The present results also show that FBS can promote closure of the defects in CE cell layers more effectively than NGF, and that addition of NGF in the presence of FBS does not further promote defect closure. Even though FBS contains NGF, these findings suggest that other components of FBS may also be involved in CE defect closure and that these other components may be more effective than NGF or may potentiate NFG’s effect [19]. It should also be noted, however, that the NGF used in this study was recombinant human NGF, while FBS contains natural, fetal NGF. This raises the possibility of a difference in the activities of the two forms of NGF in this system. In addition, the NGF present in FBS may be complexed with other components in the serum which enhances its activity.

Application of these results to the treatment of NK must be made judiciously. The present assay system lacks many of the elements that could modulate corneal healing in vivo including the corneal innervation, the tear film, systemic factors such as inflammatory mediators, and interactions between CE cells and other corneal cells [1,2,3]. NGF might be as effective, or more effective, than FBS in the more complex milieu encountered in vivo. Several other complex preparations analogous to FBS, including autologous human serum, human umbilical cord serum, human platelet-rich plasma, and human amnionic membranes, have been shown to effectively treat NK [8,12,13,14]. A systematic review of studies comparing the healing times associated with these preparations to that of NGF treatment did not reveal any convincing differences [20]. These studies, however, were done without an internal NGF comparator arm and the quality of evidence for most studies was low.

In summary, the present results suggest that the combination of bioactive components present in FBS is more effective than NGF alone in producing corneal healing in a simple model system. This raises the possibility that some of these substances may also be effective in treating NK. Additional work with more complex model systems and better-defined treatment preparations will be needed to establish this effectiveness and identify the active components. For example, a more sophisticated model system could be used to distinguish between effects of active agents on cell growth, cell migration, and cell viability. In addition, the use of heat-inactivated FBS or of other defined growth factors could help to define the active components themselves. The results of this study may encourage such work.

## Data Availability

The original contributions presented in this study are included in the article. Further inquiries can be directed to the author.
